# Effect of dried *Chlorella vulgaris* and *Chlorella* growth factor on growth performance, meat qualities and humoral immune responses in broiler chickens

**DOI:** 10.1186/s40064-016-2373-4

**Published:** 2016-06-14

**Authors:** Byoung-Ki An, Kwan-Eung Kim, Jin-Young Jeon, Kyung Woo Lee

**Affiliations:** Laboratory of Poultry Nutrition, Department of Animal Science and Technology, College of Animal Bioscience and Technology, KonKuk University, 120 Neungdong-ro, Gwangjin-gu, Seoul, 143-701 South Korea; Department of Health Food R&D, Daesang Corp., Icheon, 467-813 Korea

**Keywords:** Chlorella vulgaris, Chlorella growth factor, Growth performance, Humoral immune response

## Abstract

This experiment was carried out to investigate the effects of dried chlorella powder (*Chlorella vulgaris*; DCP) and chlorella growth factor (CGF) on growth performance, serum characteristics, meat qualities and humoral immune responses in broiler chicks. A total of 1050 day-old Ross male broiler chicks were randomly divided into 35 pens (30 chicks/pen) and subjected to one of seven dietary treatments. A non-medicated corn-soybean meal base diet was considered as negative control (NC) and added with either antibiotic (PC), three levels of DCP (NC diets added with 0.05, 0.15 or 0.5 % DCP) or two levels of CGF (NC diets added with 0.05 or 0.15 % CGF). The final body weight and daily weight gain in PC and groups fed diets with 0.15 or 0.5 % DCP were heavier (*p* < 0.001) than those of NC and CGF-treated groups. Serum total lipid concentrations were lower (*p* = 0.001) in groups fed diets with 0.5 % DCP and 0.05 or 0.15 % CGF compared with PC group. The levels of serum IgG (*p* = 0.050) and IgM (*p* = 0.010) were elevated in chicks fed diets with DCP and CGF compared with the PC or NC group. Meat qualities such as cooking loss, meat color, and pH, of edible meats were not altered by dietary treatments. Collectively, these results indicate that dietary DCP, but not CGF, exerted growth-promoting effect, and both DCP and CGF affected humoral immune response in broiler chicks.

## Background

Microalgae, microscopic unicellular organisms, can be utilized to produce a wide range of metabolites such as protein, fat, carbohydrate, vitamins and organic minerals for food and animal feeds (Becker 1994), although there was a great variation in the nutritional values of different algae meal samples (Lipstein and Hurwitz 1983). It is known that the most biotechnologically relevant microalgae are the green algae (Chlorophycea) which are widely commercialized, mainly as nutritional supplements for human beings and animals (Khan et al. 2005). Chlorella (*Chlorella vulgaris*) is a genus of single-cell green algae and is characterized by a relative ease of cultivation with high productivity and contains high contents of protein, chlorophyll, lutein and other essential micro-nutrients (Buono et al. 2014; Jeon et al. 2012). Due to high crude protein content, it is reported that dietary chlorella could replace fish meal and soybean meal by 5 and 10 % without negative effects on body weight gain and feed conversion ratio in growing chicks (Lipstein and Hurwitz 1983). Chlorella as a feed supplement has been known to have beneficial effects, such as growth, immuno-modulation, antioxidant activity and tissue rebuilding (Guzmán et al. 2001). It is reported that dietary chlorella improved growth performance, modulated immune response (Kotrbáček et al. 1994) and affected intestinal microbial diversity (Janczyk et al. 2009) in chickens.

A variety of chlorella-based products including liquid and powdered chlorella, fermented chlorella and chlorella extracts, are currently being introduced as a feed additive. Among these potential substrates, the hot water extracts from chlorella are known as chlorella growth factor (CGF) and are rich in amino acids, peptides, vitamins, minerals and nucleic acids (Merchant and Andre 2001). It has been shown that dietary CGF promoted growth and healing (Merchant and Andre 2001), stimulated immune system (An et al. 2010), and controlled the body weight and serum lipid (Hidaka et al. 2004). We have recently reported that dried chlorella powder (DCP) added into a diet of laying hens improved egg qualities and enhanced lutein contents in egg yolks (Jeon et al. 2012; An et al. 2014). The objective of present study was further explored to evaluate the two forms of chlorella as the potential candidate for alternatives to antibiotics in broiler chickens. In this study, we used DCP and CGF to see their effects on growth performance, meat quality, blood characteristics and immune parameters in broiler chicks.

## Results and discussion

The growth performance in chicks fed diets with varying levels of DCP and CGF are presented in Table 1. Dietary CGF did not affect the production traits compared with NC. As expected, PC versus NC increased final body weight. Chicks fed diets with 0.15 or 0.5 % DCP had a significantly higher final body weight compared with that of NC. During starter and grower periods, chicks in PC and 0.15 or 0.5 % DCP showed a higher (*p* < 0.05) daily body weight gain as compared with NC. This increase in daily body weight gain, especially during the grower period, was reflected in improved feed conversion ratio in chicks fed diet with antibiotics or 0.5 % DCP. There was no effect on feed intake between experimental groups. The present study indicated that dietary DCP, but not CGF, enhanced growth performance in broiler chickens. In contrast to our finding, dietary supplementation of 0.5 % biomass of chlorella did not affect final body weight in broiler chicks in study of Kotrbáček et al. (1994). On the other hand, Kang et al. (2013) reported that several chlorella-based supplements including DCP, liquid media or CGF added into the diets of broiler chicks enhanced body weight, but did not affect feed intake and feed conversion ratio. Total mortality rate for the entire period was 1.62 % (n = 17 chickens) and none of dietary treatments affected the mortality.

**Table 1:** Effect of dried chlorella powder (DCP) and chlorella growth factor (CGF) on growth performance in broiler chickens

Item	NC^1^	PC^2^	DCP			CGF		SEM^3^	*p* value
			0.05 %	0.15 %	0.5 %	0.05 %	0.15 %		
Initial BW^4^ (g/bird)	45.1	45.1	45.1	45.1	45.1	45.1	45.1	0.04	0.967
Final BW (g/bird)	1562^b^	1645^a^	1533^b^	1619^a^	1643^a^	1569^b^	1564^b^	14.06	<0.001
BW gain, g/d/bird									
1–21 days	24.0^b^	25.5^a^	23.9^b^	25.3^a^	25.7^a^	24.1^b^	24.2^b^	0.32	<0.001
22–35 days	73.7^bc^	77.8^a^	72.2^c^	76.3^ab^	77.5^a^	74.5^bc^	73.8^c^	0.77	<0.001
1–35 days	44.6^b^	47.0^a^	43.8^b^	46.3^a^	47.0^a^	44.8^b^	44.7^b^	0.41	<0.001
Feed intake (g/d/bird)									
1–21 days	38.8	40.1	39.3	40.2	41.0	39.4	39.6	0.58	0.197
22–35 days	142.9	144.9	143.8	146.2	144.3	139.8	141.8	1.73	0.215
1–35 days	81.7	83.3	82.3	83.8	83.1	80.8	81.7	0.77	0.072
Feed:gain									
1–21 days	1.62	1.57	1.65	1.59	1.60	1.64	1.63	0.03	0.484
22–35 days	1.93^ab^	1.86^b^	1.99^a^	1.92^ab^	1.86^b^	1.88^b^	1.92^ab^	0.03	0.021
1–35 days	1.83^ab^	1.77^b^	1.88^a^	1.81^b^	1.78^b^	1.80^b^	1.83^ab^	0.02	0.011

Each value represents mean of five replicates per treatment

^1^Negative control (non-medicated basal diet)

^2^Positive control (NC + virginiamycin 10 ppm)

^3^Pooled standard error of the mean

^4^Body weight

^a–c^Mean values with different superscripts within the same row differ significantly (*p* < 0.05)

In this study, dietary chlorella did not affect relative organ weights including liver, spleen, bursa of Fabricius and abdominal fat, and the relative percentage yields of breast and leg muscles (Table 2). In addition, blood parameters including albumin, total protein, GOT, total cholesterol, HDL cholesterol, triacylglycerol were not altered by dietary treatments (Table 3). However, chicks fed a diet containing 0.5 % DCP exhibited (*p* < 0.05) the lowest serum total lipid levels. Our study corroborates with previous study by Kotrbáček et al. (2013) who reported that dietary DCP did not affect the concentration of plasma triacylglycerol and cholesterol in laying hens. On the other hand, chlorella exhibited the hypocholesterolemic effect in mildly hypercholesterolemic human beings (Ryu et al. 2014) or in ovariectomized rats (Hidaka et al. 2004). Serum GOT and GPT are considered as the indicators of normal liver functions. Measurement of these enzymes as indicative of liver functionality in birds is considered to evaluate a safe inclusion levels for non-conventional feedstuffs (Diaz et al. 2003). Based on this result, DCP and CGF appeared safe and had no any adversary effects on physiological and nutritional status.

**Table 2:** Effect of dried chlorella powder (DCP) and chlorella growth factor (CGF) on relative weights of various tissues in broiler chickens

Item	NC^a^	PC^b^	DCP			CGF		SEM^c^	*p* value
			0.05 %	0.15 %	0.5 %	0.05 %	0.15 %		
Liver	2.26	2.16	2.28	2.24	2.11	2.29	2.14	0.06	0.212
Spleen	0.10	0.10	0.10	0.09	0.09	0.09	0.09	0.01	0.875
Bursa of fabricius	0.22	0.21	0.22	0.2	0.23	0.27	0.22	0.01	0.711
Abdominal fat	1.84	2.03	1.77	2.01	1.95	1.92	1.96	0.11	0.645
Breast meat	6.20	6.24	6.48	6.48	6.39	6.32	6.38	0.18	0.894
Leg meat	8.87	9.37	9.04	9.30	9.14	9.27	9.04	0.24	0.825

Each value expressed as relative percentage to body weight represents the mean of eight chicks per treatment

^a^Negative control (non-medicated basal diet)

^b^Positive control (NC + virginiamycin 10 ppm)

^c^Pooled standard error of the mean

**Table 3:** Effect of dried chlorella powder (DCP) and chlorella growth factor (CGF) on blood profiles in broiler chickens

Item	NC^1^	PC^2^	DCP			CGF		SEM^3^	*p* value
			0.05 %	0.15 %	0.5 %	0.05 %	0.15 %		
Albumin (g/dL)	1.16	1.17	1.16	1.11	1.09	1.14	1.19	0.02	0.106
Total protein (g/dL)	2.89	2.65	2.77	2.78	2.72	2.74	2.78	0.08	0.513
GOT^4^ (IU/L)	258.5	243.1	236.6	237.0	242.5	238.8	253.4	7.10	0.233
Total cholesterol (mg/dL)	117.4	127.4	120.3	120.1	109.5	114.3	116.9	4.06	0.105
HDL^5^-cholesterol (mg/dL)	96.0	106.8	97.1	93.2	86.1	96.5	94.9	4.27	0.076
Total lipid (mg/dL)	310.4^ab^	330.3^a^	314.6^ab^	308.4^ab^	252.0^c^	264.0^bc^	281.7^bc^	14.47	0.001
Triacylglycerol (mg/dL)	33.0	33.1	30.7	34.4	34.1	38.9	34.0	3.22	0.772

Each value expressed as relative percentage to body weight represents the mean of eight chicks per treatment

^1^Negative control (non-medicated basal diet)

^2^Positive control (NC + virginiamycin 10 ppm)

^3^Pooled standard error of the mean

^4^Glutamic-oxaloacetic transaminase

^5^High density lipoprotein

^a–c^Mean values with different superscripts within the same row differ significantly (*p* < 0.05)

The effect of DCP and CGF on humoral immune responses is shown in Table 4. The antibody titers against NDV and IBV in chicks fed DCP and CGF were not affected. On the other hand, the concentrations of plasma IgG were elevated in chicks fed diets containing 0.05 and 0.5 % DCP compared with those in NC or PC groups. The chicks fed diet with 0.05 and 0.15 % DCP, or 0.15 % CGF had higher concentration of plasma IgM compared with NC or PC groups. Plasma IgA was not altered by dietary treatments. It has been reported that dietary spirulina or chlorella-based products modulated host immune responses. For example, Qureshi et al. (1996) suggested that White Leghorn chicks fed a diet enriched with 1 % spirulina had higher antibody levels against sheep red blood cells (SRBC) compared with the control group. Furthermore, dietary spirulina increased serum concentration of IgG and stimulated phagocytic activity in broiler chicks (Qureshi et al. 1996). It is reported that either DCP or CGF improved immune functions in rodents (An et al. 2008) and chickens (Kotrbáček et al. 1994). Dietary chlorella enhanced the antibody productions of IgM and IgG by splenocytes and mesenteric lymphocytes in male Sprague–Dawley rats (Kanouchi 2001). Kang et al. (2013) also reported that dietary liquid chlorella media increased the concentration of plasma IgM and IgG in broiler chicks. Thus, the present study confirmed the well-established immune modulation properties of chlorella.

**Table 4:** Effect of dried chlorella powder (DCP) and chlorella growth factor (CGF) on antibody titers and the concentrations of IgG, IgA and IgM in broiler chickens

Item	NC^1^	PC^2^	DCP			CGF		SEM^3^	*p* value
			0.05 %	0.15 %	0.5 %	0.05 %	0.15 %		
NDV^4^ titer (log)	1.83	1.71	2.86	2.50	2.20	2.50	2.50	0.85	0.924
IBV^5^ titer (log)	3.29	3.00	4.13	4.57	4.00	4.75	4.86	0.51	0.076
IgA (µg/mL)	538	398	721	710	602	806	683	127.0	0.260
IgG (µg/mL)	2634^bc^	2460^c^	3814^a^	3563^abc^	3827^a^	3122^abc^	3673^ab^	359.5	0.050
IgM (µg/mL)	155^b^	152^b^	480^a^	501^a^	322^ab^	307^ab^	472a	83.0	0.010

Each value expressed as relative percentage to body weight represents the mean of eight chicks per treatment

^1^Negative control (non-medicated basal diet)

^2^Positive control (NC + virginiamycin 10 ppm)

^3^Pooled standard error of the mean

^4^Newcastle disease virus

^5^Infectious bronchitis virus

^a–c^Mean values with different superscripts within the same row differ significantly (*p* < 0.05)

In this study, the physico-chemical meat properties such as cooking loss, meat color, and pH of breast and leg muscles were not affected by dietary treatment although dietary DCP tended (*p* = 0.063) to lower the cooking loss in leg meat (Table 5). Our result is unexpected in the light of previous studies showing that fermented chlorella (Oh et al. 2015) or spirulina (Toyomizu et al. 2001) enhanced meat colors due to the efficient transfer of active carotenoids present in chlorella or spirulina. The absence of effect of both DCP and CGF on meat color cannot be explained by the inclusion levels, duration of feeding or chicken strains used. Thus, clear explanation is not readily available as we did not measure the contents of carotenoids in meat samples.

**Table 5:** Effect of dried chlorella powder (DCP) and chlorella growth factor (CGF) on meat quality in broiler chickens

Item	NC^a^	PC^b^	Chlorella powder			Chlorella growth factor		SEM^c^	*p* value
			0.05 %	0.15 %	0.5 %	0.05 %	0.15 %		
Cooking loss									
Breast meat	28.2	26.6	27.1	26.1	26.5	27.5	26.9	0.88	0.683
Leg meat	35.5	35.3	31.8	32.7	34.4	35.5	35.6	1.06	0.063
Color of breast meat									
L*	58.8	59.2	60.3	58.6	58.9	58.4	58.5	0.86	0.781
a*	0.96	0.67	1.24	0.57	0.87	0.76	0.99	0.24	0.505
b*	8.81	10.28	7.89	8.15	7.86	8.98	9.08	0.63	0.107
Color of leg meat									
L*	54.2	54.5	55.5	52.7	54.4	55.1	53.0	0.92	0.286
a*	3.16	2.53	2.84	3.47	3.06	3.11	3.05	0.37	0.715
b*	7.66	9.28	9.01	7.12	8.63	7.93	8.80	0.61	0.136
pH									
Breast meat	5.69	5.70	5.69	5.74	5.68	5.79	5.72	0.04	0.578
Leg meat	5.85	5.89	5.87	5.94	5.86	5.91	5.89	0.04	0.585

Each value expressed as relative percentage to body weight represents the mean of eight chicks per treatment

^a^Negative control (non-medicated basal diet)

^b^Positive control (NC + virginiamycin 10 ppm)

^c^Pooled standard error of the mean

## Conclusions

It is concluded that dietary DCP, but not CGF, exhibited growth-promoting effect and both DCP and CGF enhanced humoral immune responses, reflecting potential alternative substances to replace in-feed antibiotics.

## Methods

All animal care procedures were approved by Institutional Animal Care and Use Committee in Konkuk University.

### Bird husbandry, study design and diets

Dried *Chlorella vulgaris* powder (DCP) and chlorella growth factor (CGF) were provided by Daesang Corp. (Icheon, Korea). The DCP, the powdered form of dried chlorella, was made from liquid chlorella obtained from heterotrophic tank. The CGF was prepared by spray-drying and subsequently lyophilizing the hot-water extracts of DCP.

On the day of hatch, Ross 308 male broiler chicks were received from a local hatchery. They were weighed individually and randomly assigned into 35 pens with 30 chicks per pen. In total, 1050 chicks were housed on rice husks as a bedding material with 23/1 light/dark cycle of 20 lx intensity throughout the experimental period and fed one of seven diets for 35 day of rearing period; two control diets with or without antibiotics, three diets containing 0.05, 0.15 or 0.5 % DCP, and two diets containing 0.05 or 0.15 % CGF, respectively. The corn-soybean meal base diet (Table 6) was used as negative control (NC). The NC diet was then added with virginiamycin (10 mg/kg of diet; PC) or different doses of DCP and CGF. The chicks were allowed to have free access to diet and water. Temperature of the facility was set at 33 °C for the first 3 days, gradually decreased to reach 23 °C and kept thereafter until the end of experiment. The experimental diets were freshly added daily and feed intake of each pen was recorded weekly. The body weight on a pen basis was recorded weekly and used to calculate feed conversion ratio. All chicks were intramuscularly vaccinated with Newcastle disease virus (NDV) and infectious bronchitis virus (IBV) twice at 14 and 28 days.

**Table 6:** Formula and chemical compositions of the basal diets

Ingredients (%)	Starter (0–20 days)	Grower (21–35 days)
Yellow corn	53.04	52.79
Wheat	7.00	6.00
Rice polishing	3.00	2.00
Soybean meal	17.34	21.13
Rapeseed meal	3.50	3.00
Corn gluten meal	5.98	5.50
Fish meal	3.00	2.80
Animal fat	3.84	3.31
Vit.-min. mixture^a^	0.20	0.20
NaHCO_3_	0.09	0.10
l-lysine HCl, 78 %	0.42	0.33
dl-Methionine, 99 %	–	0.18
l-Threonine, 99 %	0.03	–
Dicalcium phosphate	1.01	1.31
Limestone	1.16	0.94
Choline-Cl, 50 %	0.05	0.06
Salt	0.30	0.30
Anticoccidial^b^	0.05	0.05
Total	100	100
Calculated values		
MEn (kcal/kg)	3160	3220
Crude protein (%)	20.5	19.5
Ca (%)	1	0.9
Avail. P (%)	0.4	0.35
Total lysine (%)	1.1	1.01
Total TSAA (%)	0.93	0.73

^a^Vit. Min. mixture provided the following nutrients per kg of diet: vitamin A, 18,000 IU; vitamin D_3_, 3750 IU; vitamin E, 30 IU; vitamin K_3_, 2.7 mg; vitamin B_1_, 3.0 mg; vitamin B_2_, 9.0 mg; vitamin B_6_, 4.5 mg; vitamin B_12_, 30.0 mg; niacin, 37.5 mg; pantothenic acid, 15 mg; folic acid, 1.5 mg; biotin, 0.07 mg; Fe, 75.0 mg; Zn, 97.5 mg; Mn, 97.5 mg; Cu, 7.5 mg; I, 1.5 mg; Se, 0.2 mg

^b^Maduramicin (5 mg per kg of the active ingredient in the complete feed)

### Sampling

At 35 days, eight chicks per treatment were randomly selected, weighed individually and sampled blood after euthanasia by cervical dislocation. Serum samples were obtained by gentle centrifugation and stored at −20 °C prior to the analysis. Immediately after blood sampling, liver, spleen, abdominal fat, bursa of Fabricius, and left breast and boneless and skinless whole leg meats were removed and weighed. Organ and meat weights were expressed as grams of organ per 100 g body weight. The breast and leg meats were then chilled for 30 min in ice water and kept on ice prior to the measurement of meat quality.

### Measurement of blood parameters

The concentrations of total cholesterol, high density lipoprotein-cholesterol (HDL-cholesterol) and the activity of glutamic-pyruvic transaminase (GOT) in serum samples were measured by colorimetric methods using commercial cholesterol diagnostic kits (Cholesterol E kit and HDL-cholesterol kit, Youngdong Medical Co., Korea) and GOT-GPT test kit (GOT-GPT assay kit, Youngdong Medical Co., Korea). The other blood profiles, including albumin, total protein, total lipid and triacylglycerol were measured according to the colorimetric method using biochemical analyzer (Hitachi modular system, Hitachi Ltd., Tokyo, Japan).

### Measurement of humoral immune response

Serum samples were analyzed for anti-NDV and anti-IBV antibody titers by ELISA with commercial kits, following the manufacture’s direction (IDDEX Laboratory, Inc., ME). The concentrations of serum IgA, IgG and IgM were measured by commercial IgA, IgG, and IgM kits (BETHYL Laboratories, Inc. USA) as described elsewhere (Kang and Kim 2015).

### Measurement of meat qualities

The breast and leg meats were used to measure cooking loss, pH and meat color. To determine the cooking loss, 60 g of each meat was boiled individually in a polyethylene bag immersed in 75 °C water bath (C-WEB, Changshin Co., Korea) for 30 min and cooled at room temperature for 30 min. We did not measure the core temperature of meats and 30 min was the complete time of immersing samples in water both. The cooking loss was calculated from the difference in weights of uncooked and cooked meats. The pH values of breast and leg meats were estimated in triplicate with a pH meter (Model 340, Mettler-Toledo, Switzerland). Briefly, 1 g of meat sample was cut into small pieces and homogenized with 9 mL of distilled water for 1 min in an Ultra-Turrax (Model No. T25, Janken and Kunkel, Germany). The instrumental color of fresh meats, including lightness (L*), redness (a*) and yellowness (b*), was measured by a reflectance colorimeter (CR 210, Minolta, Japan) using illuminant source C. Color was measured in triplicate on the bone-side surface of each sample. The colorimeter was calibrated throughout the study using a standard white ceramic tile.

### Statistical analysis

Pen was considered as the experimental unit for growth performance. The individual bird was considered experimental unit for the rest measurements. All data were analyzed by the GLM procedure of the SAS (SAS 2002), and significant differences of obtained means were determined using Duncan’s multiple range tests at the level of *p* < 0.05.
